# Gene Expression Profiling Reveals Fundamental Sex-Specific Differences in SIRT3-Mediated Redox and Metabolic Signaling in Mouse Embryonic Fibroblasts

**DOI:** 10.3390/ijms25073868

**Published:** 2024-03-30

**Authors:** Robert Belužić, Ena Šimunić, Iva I. Podgorski, Marija Pinterić, Marijana Popović Hadžija, Tihomir Balog, Sandra Sobočanec

**Affiliations:** Laboratory for Metabolism and Aging, Division of Molecular Medicine, Ruđer Bošković Institute, 10000 Zagreb, Croatia; ena.simunic@irb.hr (E.Š.); iskrinj@irb.hr (I.I.P.); mpinter@irb.hr (M.P.); mhadzija@irb.hr (M.P.H.); balog@irb.hr (T.B.); ssoboc@irb.hr (S.S.)

**Keywords:** Sirtuin 3, sex differences, mouse embryonic fibroblasts, Hif-1α, integrated stress response, unfolded protein response

## Abstract

Sirt-3 is an important regulator of mitochondrial function and cellular energy homeostasis, whose function is associated with aging and various pathologies such as Alzheimer’s disease, Parkinson’s disease, cardiovascular diseases, and cancers. Many of these conditions show differences in incidence, onset, and progression between the sexes. In search of hormone-independent, sex-specific roles of Sirt-3, we performed mRNA sequencing in male and female Sirt-3 WT and KO mouse embryonic fibroblasts (MEFs). The aim of this study was to investigate the sex-specific cellular responses to the loss of Sirt-3. By comparing WT and KO MEF of both sexes, the differences in global gene expression patterns as well as in metabolic and stress responses associated with the loss of Sirt-3 have been elucidated. Significant differences in the activities of basal metabolic pathways were found both between genotypes and between sexes. In-depth pathway analysis of metabolic pathways revealed several important sex-specific phenomena. Male cells mount an adaptive Hif-1a response, shifting their metabolism toward glycolysis and energy production from fatty acids. Furthermore, the loss of Sirt-3 in male MEFs leads to mitochondrial and endoplasmic reticulum stress. Since Sirt-3 knock-out is permanent, male cells are forced to function in a state of persistent oxidative and metabolic stress. Female MEFs are able to at least partially compensate for the loss of Sirt-3 by a higher expression of antioxidant enzymes. The activation of neither Hif-1a, mitochondrial stress response, nor oxidative stress response was observed in female cells lacking Sirt-3. These findings emphasize the sex-specific role of Sirt-3, which should be considered in future research.

## 1. Introduction

In mammals, including humans, sex differences go beyond purely anatomical differences. Males and females differ in their hormonal status, physiological responses, susceptibility to diseases (e.g., autoimmune diseases, cardiovascular diseases and certain cancers), and life expectancy, with females living longer than males [1,2,3,4]. Furthermore, metabolic homeostasis, a cornerstone of physiological balance, is controlled by different regulatory mechanisms in males and females [5]. Although many health-related sex differences have decreased in recent years due to lifestyle changes and advances in healthcare, preclinical biomedical research should take sex factors into account to produce scientific knowledge that is relevant to both sexes. Sex-biased asymmetry in the research data is mainly caused by the tendency to exclude female rodents from study designs because it is often assumed that variability increases due to the female reproductive cycle [6]. However, such claims have been disputed through extensive meta-analyses [7,8]. Therefore, it is clear that defining sex as an equally weighted factor in biomedical research is crucial for an unbiased approach to both data generation and interpretation. Consistent with this claim, in our previous study, we analyzed sex-specific responses to a high-fat diet (HFD) and Sirt-3 status. Sirt-3, a mitochondrial deacetylase [9], plays a crucial role in maintaining cellular health. By activating or suppressing a large number of target proteins, it integrates various metabolic processes, including energy production and antioxidant defense [10,11]. Through protein deacetylation, Sirt-3 improves mitochondrial function, promotes efficient energy conversion, and reduces oxidative stress, thereby contributing to metabolic flexibility and affecting glucose and lipid metabolism. The involvement of Sirt-3 in stress response pathways helps cells adapt to changing conditions. It also plays a role in longevity and age-related diseases, making it an important target for research into interventions that could improve cellular resilience and potentially extend lifespan. We observed significant differences between Sirt-3 WT and KO mice in response to an HFD, with a subset of those differences also being sex-dependent [12,13]. These parameters include hepatic lipid accumulation and glucose uptake, protein oxidative damage, antioxidant response, and mitochondrial respiration rate. This was accompanied by changes in the expression levels of a group of genes involved in lipid metabolism and oxidative stress. To elaborate on these findings and identify the genetic networks underlying the sex-specific response, we performed RNA sequencing on Sirt-3 WT and KO male and female MEFs. Earlier studies focused on male animals or specific pathologies [14,15,16,17]. Here, we provide an overview of the transcriptomic landscape in relation to sex and Sirt-3 status and then focus on the main findings. MEFs, as a cellular model for fundamental and hormone-independent sex differences, were chosen because we aimed to detect sex-specific effects independent of hormonal status. At this early developmental stage, sexual dimorphism is not well-established, so MEFs should be advantageous for understanding fundamental cellular processes without the added complexity of hormonal interactions. Several cellular signaling pathways were affected by the loss of Sirt-3 in a sex-specific manner, with the effects of sex on hypoxia-inducible factor (Hif-1α) signaling being the most striking. The Hif-1α signaling pathway stimulates cellular adaptation to low oxygen levels by promoting the expression of a number of genes involved in glycolysis, angiogenesis and cellular survival mechanisms. While Sirt-3 has been shown to suppress Hif-1α [18], we show for the first time that Sirt-3 KO-induced activation of the Hif-1α axis is mainly restricted to male MEFs. Male-specific activation of the Hif-1α pathway is accompanied by a parallel integrated stress response (ISR), a highly conserved signaling pathway by which cells adapt to various stressors. While both sexes exhibit the ER-unfolded protein response (UPR) as a consequence of the loss of Sirt-3, mitochondrial and oxidative stress are much more pronounced in male MEFs, while female cells stay unaffected. The activation of those responses leads to male-specific major metabolic shifts. Given the importance of Hif-1α and ISR metabolic pathways, our findings once again underscore the importance of including sex as a variable in biomedical research.

## 2. Results

**Figure 1:**
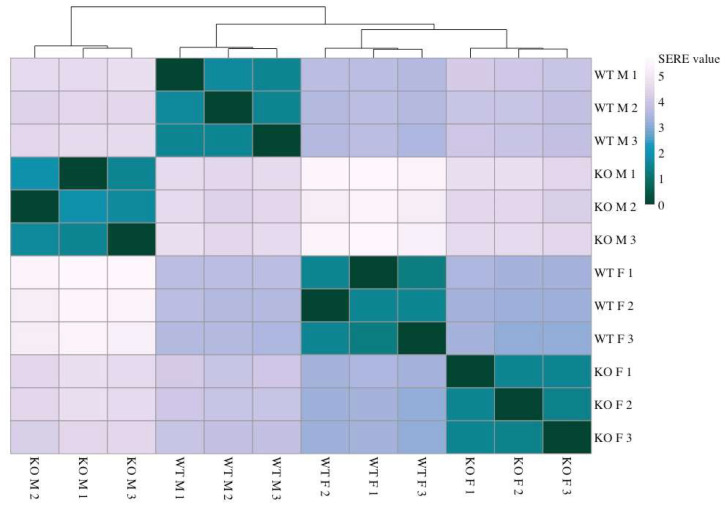
Heatmap representing distances between samples based on SERE (simple error ratio estimate) values. Higher values indicate a larger distance between samples. Lower SERE values indicate more similarity.

Samples were clustered by calculating simple error ratio estimate (SERE) coefficients as an estimate of distance among experimental groups. The global pattern of gene expression showed significant differences between both sexes and genotypes, with male MEFs modulating the expression of more genes than female MEFs as a consequence of loss of Sirt-3 function. The samples were divided into four clearly defined clusters based on their gene expression signatures (Figure 1).

The samples are well grouped by both sex and Sirt-3 status, and the difference in global gene expression between sexes is larger in Sirt-3 KO than between WT samples. The gap between WT and KO male MEFs (SERE value 4.6) was also significantly greater than that between WT and KO female MEFs (SERE value 3.4). As expected, male and female WT MEFs show distinct gene expression profiles. Importantly, the difference between male and female KO cells is significantly larger than that between male and female WT MEF suggesting the sex-specific contributing mechanisms. A sex-dependent response to Sirt-3 KO is also evident from the PCA plot (Supplementary Figure S1), with the separation between male and female KO MEFs being much larger than that between WT MEFs. When comparing gene expression between the two genotypes (WT and KO) disregarding the effect of sex, we were able to identify a total of 2714 differentially expressed genes (DEGs). This is similar to the number of DEGs detected between WT male and female (2527) MEFs, meaning that sex differences between KOs could be masked by inherent sex-specific gene expression pattern differences between WT male and female MEFs. A less stringent adjusted *p* value (*p*_adj_) of 0.05 was chosen because of the large number of proposed Sirt-3 targets, implying modulation of many Sirt-3-dependent cellular pathways that are expected to be regulated by subtle changes in gene expression. The high number of DEGs between WT male and female MEFs means that, when comparing all WT with all KO samples, each group effectively consists of two significantly different subgroups in comparison. Therefore, we decided to compare male KO vs. WT and female KO vs. WT gene expression separately. Then, sex-independent DEG sets were generated by intersecting male and female DEG sets and sex-specific DEGs by subtracting them in both ways.

### 2.1. Sirt-3-Dependent Changes in Gene Expression

**Figure 2:**
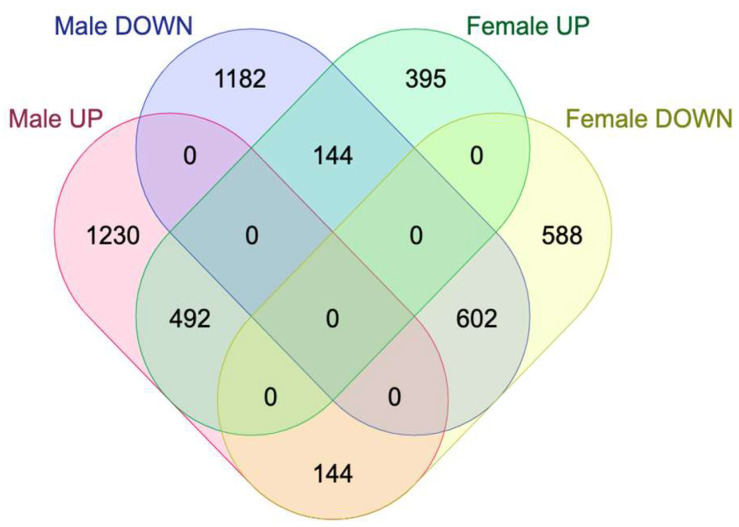
Venn diagram representing the numbers of DEGs detected in male, female or both male and female KO MEFs.

We discovered 1382 DEGs common to both male and female KO MEFs compared to WT MEFs of the same sex (Figure 2). Inherent to enrichment analyses, many reported pathways are not relevant for the specific experimental model, are not informative, or are beyond the scope of this article.

Some pathways are related to known and proposed functions of Sirt-3, such as lipid metabolism and cellular redox balance. Thus, loss of Sirt-3 affected phosphatidylcholine and cholesterol biosynthesis, fatty acid metabolism, and the oxidative stress response (Table 1). Sirt-3 is known to promote fatty acid oxidation [19] and support mitochondrial respiratory function [11]. While these results are generally valid, no significant differences between subgroups, i.e., sexes, can be detected with this commonly used averaged approach.

### 2.2. Sex-Specific Changes in Gene Expression

**Figure 3:**
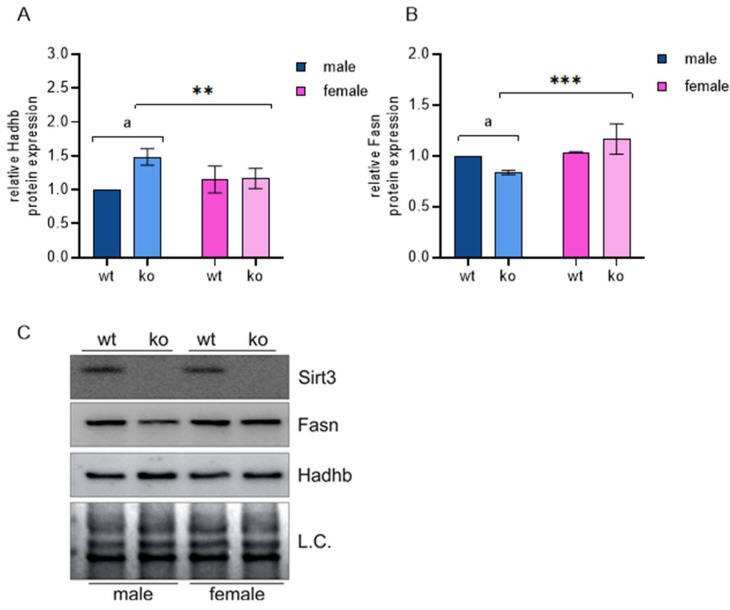
Male Sirt-3 KO MEFs show an inverse expression pattern of Hadhb and Fasn proteins. (**A**) Graphical display of the averaged densitometry values for Hadhb protein expression. Sirt-3 × sex interaction: F(1, 4) = 30.199, *p* = 0.005, partial η^2^ = 0.883; the simple effect of Sirt: F(1, 4) = 61.747, partial η^2^ = 0.939; ^a^ *p* < 0.01, male WT vs. KO; the simple effect of sex: F(1, 4) = 48.120, partial η^2^ = 0.923; ** *p* < 0.01, female KO vs. male KO. (**B**) Graphical display of the averaged densitometry values for Fasn protein expression. Sirt-3 × sex interaction: F(1, 4) = 32.895, *p* < 0.01, partial η^2^ = 0.892; the simple effect of Sirt-3: F(1, 4) = 114.632, partial η^2^ = 0.966; ^a^ *p* < 0.001, male WT vs. male KO; the simple effect of sex: F(1, 4) = 107.789, partial η^2^ = 0.964; *** *p* < 0.001, female KO vs. male KO. (**C**) Representative immunoblot of Sirt3, Fasn, and Hadhb protein expression. Two-way ANOVA with post hoc Bonferroni correction. L.C., loading control. The results are presented as the mean ± SD.

Next, we focused on defining male- and female-specific altered pathways. We generated two gene sets containing DEGs detected only in male or only in female KO MEFs. Apart from several receptor-mediated pathways, the outputs include changes in glycolysis, the pentose-phosphate pathway, and lipid and cholesterol biosynthesis, which are indicative of major sex-specific metabolic shifts. Therefore, we investigated the expression of genes involved in these processes. As shown in Table 2 and Table 3, Sirt-3 KO induces the expression of major glycolytic genes specifically in male MEFs. This includes muscle-type phosphofructokinase 1 (*Pfkm*) and phosphofructokinase 2 (*Pfkfb2*), key regulators of glycolytic flux [20]. Pfkfb2 activates Pfkm through formation of its allosteric activator, fructose 2,6-bisphosphate. Phosphorylation of fructose 6-phosphate to fructose 1,6-bisphosphate by Pfkm is the rate-limiting step in glycolysis, and cells control this flux through regulation of phosphofructokinase levels. This points to the major male-specific shift in metabolism to aerobic glycolysis as a result of the loss of Sirt-3. Another significant effect pronounced in male KO MEFs is the upregulation of the pentose-phosphate pathway (PPP). The rate-limiting step in the PPP is the conversion of glucose-6-phosphate to 6-phosphogluconolactone by glucose-6-phosphate dehydrogenase (G6pdx), which can be controlled at the transcriptional level by substrate availability or allosterically by NAD^+^. Regarding the TCA cycle, an increase in pyruvate dehydrogenase kinase (Pdk-1), an inhibitor of pyruvate dehydrogenase (Pdh), is observed in both sexes but is much more pronounced in male MEFs. Inhibition of Pdh leads to an accumulation of pyruvate, which can be further metabolized to lactate by lactate dehydrogenase. This is supported by a strong upregulation of lactate dehydrogenase (Ldhb) only in male KO MEFs. Sirt-3 is considered to most strongly affect the TCA cycle and fatty acid (FA) metabolism [21]. Many of these effects may not be due to changes in gene expression but to the modulatory activity of Sirt-3. However, male KO MEFs show a slight decrease in acetyl-CoA carboxylase (*Acaca*), which catalyzes the carboxylation of acetyl-CoA to malonyl-CoA as the first step of FA synthesis. In addition, male KO MEFs exhibited upregulation of solute carrier family 27, member 4 (*Slc27a4, Fatp4)*, suggesting that male KO MEFs switched from ATP use for the synthesis of FAS to ATP-producing FA β-oxidation. Such an effect was not observed in female MEF. This metabolic shift is further confirmed by a decrease in FA synthase (Fasn) and an increase in hydroxyacyl-CoA dehydrogenase trifunctional multienzyme complex subunit beta (*Hadhb,* a key enzyme in beta oxidation) at the protein level in male KO MEFs, as shown by a Western blot (Figure 3, Supplementary Figures S2 and S7). Female KO MEFs show the opposite behavior, increasing Acaca transcription and Fasn at both the mRNA and protein levels, thereby increasing the rate of fatty acid synthesis.

### 2.3. OXPHOS and MEF Energy Status

The regulation of ATP generation through oxidative phosphorylation (OXPHOS) is a complex system of tuning OXPHOS-related gene transcription, protein synthesis, and import to mitochondria, posttranslational modifications to control their activity, allosteric control by substrates/products, and the number and size of mitochondria [22,23,24,25,26,27]. We used an OXPHOS-related gene list from the KEGG database to detect potential differences in the transcript levels between male and female Sirt-3 KO MEFs compared to their corresponding WT controls. At the transcription level, we could not detect any significant differences in the expression of major OXPHOS regulators. On the other hand, we show that male KO MEFs are indeed energy-depleted, which is reflected by the increase in protein kinase AMP-activated catalytic subunit alpha 2 (*Prkaa2*). *Prkaa2* is a gene that encodes the catalytic subunit of AMP-activated protein kinase (AMPK), a key cellular energy sensor that plays a crucial role in regulating cellular energy balance [28,29]. Energy deficit in males is supported by our previous study, which showed a decrease in C1-driven oxygen consumption in WT and KO male mice compared to WT and KO females [12].

**Figure 4:**
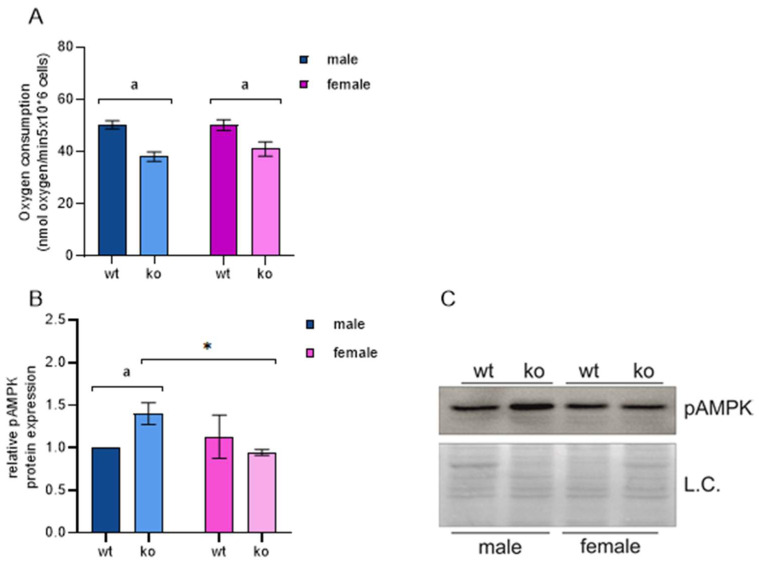
Sirt-3 KO increases AMPK phosphorylation in a male-specific manner. (**A**) Complex 1 (C1)-driven ADP-stimulated respiration (state 3). Sirt × sex interaction: F(1, 20) = 3.287, *p* = 0.085, partial η^2^ = 0.141; the main effect of Sirt-3: F(1, 20) = 156.339, partial η^2^ = 0.887; ^a^ *p* < 0.001, male WT vs. male KO, female WT vs. female KO. (**B**) Graphical display of the averaged densitometry values for pAMPK protein expression. Sirt-3 × sex interaction: F(1, 4) = 8.322, *p* < 0.05, partial η^2^ = 0.675; the simple effect of Sirt-3: F(1, 4) = 7.781, partial η^2^ = 0.660; ^a^ *p* < 0.05, male WT vs. male KO; the simple effect of sex: F(1, 4) = 10.068, partial η^2^ = 0.716; * *p* < 0.05, female KO vs. male KO. (**C**) Representative immunoblot of pAMPK protein expression. Two-way ANOVA with post hoc Bonferroni correction. L.C., loading control. The results are presented as the mean ± SD.

Both AMPK mRNA and its phosphorylation are increased specifically in male KO MEFs (Table 2, Figure 4, Supplementary Figure S3). AMPK is induced by a low ATP/ADP ratio and modulates the activity of a large number of target proteins to restore proper cellular energy status [28]. As Sirt-3 is known to support proper mitochondrial function, C1-driven respiration was impaired both in male and female MEFs (Figure 4A), as expected. On the other hand, we observed that only male KO MEFs are held in an energy-deficient state, while female MEFs are able to maintain the proper ATP/ADP ratio. A decrease in ATP production through OXPHOS induces AMPK, which suppresses anabolic pathways such as fatty acid synthesis and supports energy-producing pathways such as beta-oxidation. The results described in a previous chapter confirm this finding through downregulation of *Acaca*/*Fasn* and upregulation of *Slc27a4* in male KO MEFs. Thus, the core metabolic pathway equilibrium in male MEF is highly dependent on Sirt-3, while female MEFs can compensate for the loss of Sirt-3 function and consequential decrease in OXPHOS through yet unknown mechanism(s).

Taken together, the male-specific metabolic effects of Sirt-3 KO include a shift to glycolysis and downregulation of the TCA cycle, along with OXPHOS and compensatory ATP production from fatty acids. This phenomenon can occur when primary nutrients are scarce, not available due to downregulation of relevant transporters, or as a consequence of mitochondrial function disruption. Since nutrients are available to cultured cells and transporter downregulation was not observed, the latter appears to be the most likely explanation.

**Figure 5:**
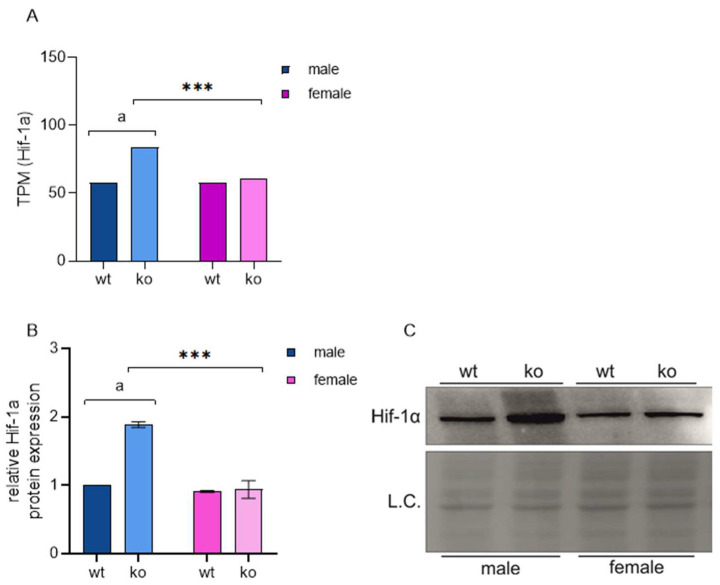
The KO of Sirt-3 leads to a male-specific increase in both Hif-1α gene and protein expression. (**A**) Graphical display of Hif-1α gene expression in TPM values. Male KO MEFs show a significant increase compared to male WT MEFs (log_2_FC = 0.53 ± 0.047, ^a^ *p*_adj_ = 6.8 × 10^−27^) and to KO female MEFs (log_2_FC = 0.463 ± 0.042, *** *p*_adj_ = 1.7 × 10^−28^). TPM—Transcripts per Million; log_2_FC—log_2_ scaled fold change ± S.E.; *p*_adj_—adjusted *p* value. (**B**) Graphical display of the averaged densitometry values for Hif-1α protein expression. Sirt-3 × sex interaction: F(1, 4) = 79.853, *p* < 0.001, partial η^2^ = 0.952; the simple effect of Sirt-3: F(1, 4) = 90.202; partial η^2^ = 0.977; ^a^ *p* < 0.001, male WT vs. male KO; the simple effect of sex: F(1, 4) = 194.903, partial η^2^ = 0.980; *** *p* < 0.001, female KO vs. male KO (**C**) Representative immunoblot of Hif-1α protein expression. Two-way ANOVA with post hoc Bonferroni correction. L.C., loading control. The results are presented as the mean ± SD.

Sirt-3 is known to maintain mitochondrial homeostasis. Therefore, its absence is expected to affect normal respiration and force cells to compensate by the abovementioned mechanisms. However, our results show for the first time that this effect is sex-specific. Loss of Sirt-3 leads to an increase in mitochondrial ROS due to an inefficient electron transport chain [30]. High ROS levels mimic the hypoxic state at physiological pO_2_, a phenomenon termed ‘pseudohypoxia’. First coined to describe a hypoxia-like phenomenon in diabetes, pseudohypoxia is now generally defined as a cellular response that resembles that to hypoxia but occurs under normoxic conditions [31]. The cellular response to hypoxic conditions is mediated mainly by hypoxia-induced factor 1a (Hif-1α). Hif-1α is a well-known, ubiquitously expressed transcription factor with over 1000 known target genes that regulate various cellular processes, such as energy metabolism, proliferation, apoptosis, stem cell maintenance and tissue development. Most importantly, Hif-1α is a major regulator of the cellular response to hypoxia or pseudohypoxia. The stabilization of Hif-1α in the absence of Sirt-3 has been described previously [18,32] and is mostly considered in the context of the tumor microenvironment and the Warburg effect. However, these studies did not provide a complete gene expression signature and were limited to a small number of genes/proteins. Additionally, differences between sexes were not considered. Here, we show that male KO MEFs accumulate significantly more Hif-1α than female KO MEFs. While Hif-1α levels in WT male and female MEFs remain similar, the KO of Sirt-3 leads to a male-specific increase in both Hif-1α mRNA and protein (Figure 5, Supplementary Figure S4).

Under normoxic conditions, specific prolyl residues of Hif-1α are hydroxylated by prolyl hydroxylases (PHDs). Hydroxylated Hif-1α is recognized by VHL (von Hippel–Lindau) protein, and this interaction leads to ubiquitination and subsequent proteasomal degradation of Hif-1α [33]. In contrast, low oxygen levels and ROS inhibit PHDs, leading to Hif-1α accumulation. After being translocated to the nucleus, Hif-1α forms an active complex with Hif-1β. The Hif-1α/β complex then binds to HRE (hypoxia-responsive elements) in target gene promoters. The male-restricted Hif-1α response reflects higher ROS generation in male MEFs upon Sirt-3 loss. We therefore extracted expression data for genes related to oxidative stress management. Among superoxide dismutases, superoxide dismutase 1 (*Sod-1*) was expressed at the highest level, followed by *Sod-2* and *Sod-3*. Significant differences detected between sexes were an increase in *Sod-1* in female KO MEFs and extracellular *Sod-3* in male KO MEFs, although the overall expression of *Sod-3* was very low. Furthermore, catalase was increased only in male KO MEFs (log_2_FC = 0.26, *p*_adj_ = 0.006). Among glutathione peroxidases (GPXs), male KO MEFs specifically upregulated *Gpx-7*, an ER-associated enzyme responsible for ROS and lipid peroxide detoxification in the ER. Mitochondrial *Gpx-4* was also increased in male MEFs, but this increase was not statistically significant. Glutathione S-transferases *Gstm-1, Gstm-5* and *Gstz-1*, quinone reductase *Nqo1* and thioredoxin inhibitor *Txnip* were upregulated specifically in male MEFs. On the other hand, female KO MEFs expressed higher levels of a subset of oxidative stress protective genes, such as *Gpx-8*, *Gsto-1*, *Sod-1, Nxn* (nucleoredoxin—also a Wnt signaling inhibitor) and the redox-sensitive chaperone *Park-7.* Although the pattern of sex differences in ROS detoxifying enzymes is not clear, female MEFs appear to cope more efficiently with the increase in ROS generation induced by loss of Sirt-3, thereby avoiding a significant Hif-1α response. Females have been previously proposed to be less sensitive to oxidative stress [34,35], as also excellently reviewed in [36]. Our data suggest that female MEFs are inherently more efficient at eliminating ROS than male cells and are therefore less dependent on Sirt-3 function, whereas male KO MEFs are forced to sustain a response to chronic ROS overproduction. This is also supported by the growth curves for male and female KO MEFs (internal data), where female MEFs exhibited faster growth and viability along with lower mitochondrial ROS levels. In brief, Sirt-3 deficiency affects mitochondrial function and induces a pseudohypoxic state and ROS increase with a concomitant Hif-1α increase specifically in male KO MEFs. Compromised respiration inevitably leads to the generation of excess ROS, resulting in cellular stress and a corresponding response. Given the variety of Sirt-3 targets, effects beyond oxidative stress can also be expected, such as mitochondrial protein folding or nutrient utilization due to metabolic shifts. Cells respond to these and other stressors through the ISR [37]. Therefore, we investigated the expression of the main ISR regulators and their targets.

### 2.4. Sirt-3 Loss Leads to Male-Restricted, Atf-4-Mediated ISR

**Figure 6:**
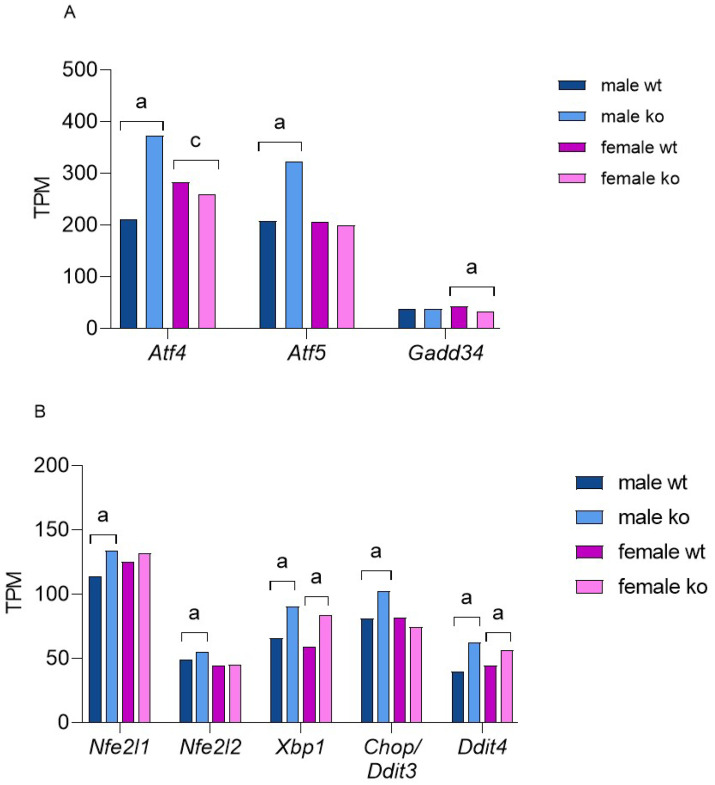
TPM values of the main ISR regulators and effectors in male and female Sirt-3 KO MEFs. (**A**) Graphical display of gene expression of *Atf4*: ^a^ *p* < 0.001, male WT vs. male KO; ^c^ *p* < 0.05, female WT vs. female KO; *Atf5*: ^a^ *p* < 0.001, male WT vs. male KO; Gadd34: ^a^ *p* < 0.001, female WT vs. female KO. (**B**) Graphical display of gene expression of *Nfe2I1:* ^a^ *p* < 0.001, male WT vs. male KO; *Nfe2I2*: ^b^ *p* < 0.01, male WT vs. male KO; *Xbp1*: ^a^ *p* < 0.001, male WT vs. male KO, female WT vs. female KO; *Chop/Ddit3*: ^a^ *p* < 0.001, male WT vs. male KO; *Ddit4*: ^a^ *p* < 0.001, male WT vs. male KO, female WT vs. female KO; (exact TPM and *p*_adj_ values can be found in Supplementary Table S1). TPM—Transcripts per Million.

Sirt-3 KO induces cellular stress, primarily through impaired mitochondrial function and excessive ROS production, putting the cell in a pseudohypoxic state. It is plausible that the changes in cell metabolism described above act as secondary stressors, such as specific nutrient deprivation, aberrant signaling or damage to other organelles. Eukaryotic cells react to various stresses through the ISR, a highly conserved mechanism. The activation of ISR serves to restore cellular homeostasis and results in a global decline in Cap-dependent translation and sustained translation of ISR-specific mRNAs. Different stressors activate a specific kinase, such as protein kinase R (PKR)-like endoplasmic reticulum kinase (Perk), protein kinase R (Pkr), hepatic heme-regulated inhibitor (Hri) or serine/threonine-protein kinase general control nonderepressible 2 (Gcn2). Oxidative stress activates all four of them [38,39,40,41]. Activated kinases then phosphorylate eukaryotic initiation factor 2 (eIF-2α), and phosphorylated eIF-2α preferably translates activating transcription factor 4 (Atf-4), activating transcription factor 5 (Atf-5), C/EBP homologous protein (*Chop*, *Ddit-3* gene), and growth arrest and DNA damage-inducible protein (*Gadd-34, Ppp1r15a* gene) thus activating the ISR program. Gadd-34 dephosphorylates eIF-2α, creating a negative feedback loop to blunt the stress response. The main ISR effector is Atf-4, which induces the expression of a number of genes in a pattern that optimizes the cellular response to a specific type of stress [37,42,43]. Here, we show that Atf-4-mediated ISR in Sirt-3 KO MEFs is highly male-specific. Figure 6 shows the expression of the main ISR regulators and transcription factors.

In addition to *Atf-4*, male KOs significantly increased the expression of *Atf-5* and CCAAT-enhancer-binding protein homologous protein (*Chop/Ddit-3*), key regulators of mitochondrial UPR [44]. The data indicate that the loss of Sirt-3 activates the Atf-4/Ddit-3 UPR^mt^ exclusively in male MEFs. Nuclear factor erythroid 2-related factor 2, *Nfe2l2 (Nrf-2*), is induced by oxidative stress and plays a key role in activating cellular defense against oxidative damage [45]. Recent research has proven that *Nrf-2* is induced by *Atf-4* as an integral part of oxidative stress-induced ISR [46]. Figure 6 shows that Sirt-3 KO induces an *Atf-4*/*Nrf-2*-mediated response to oxidative stress only in male MEFs. On the other hand, *Xbp-1*, a key transcription factor in the cellular response to endoplasmic reticulum (ER) unfolded protein stress [47], is induced in KOs of both sexes. Finally, both male and female KO MEFs upregulated *Ddit-4* (regulated in development and DNA damage response 1; *REDD-1*), which can be induced by *Atf-4, Hif-1α and Xbp-1* in the presence of various stressors, including the accumulation of misfolded proteins in the ER. The *Ddit-4* increase in both female and male Sirt-3 KOs further confirms that loss of Sirt-3 leads to ER proteotoxic stress that is both sex- and *Atf-4*-independent.

**Figure 7:**
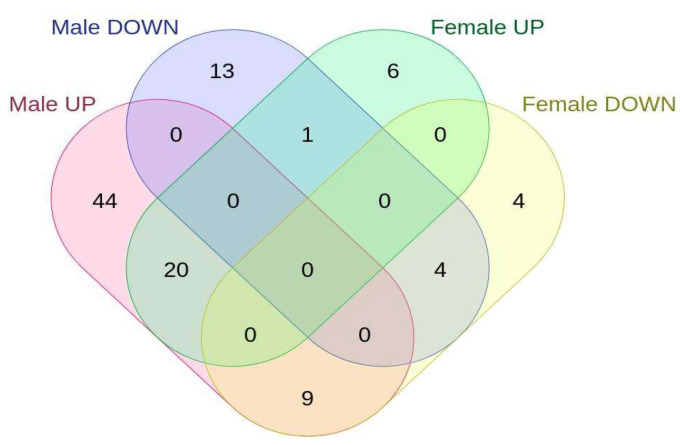
Venn diagram representing differentially expressed Atf-4 targets in Sirt-3 WT and KO male and female MEFs.

To explain the nature of male KO-restricted ISR, we analyzed the expression of known Atf-4 targets. The target gene list was taken from an excellent systematic review [48]. We detected a total of 91 differentially expressed Atf-4 targets in male MEFs (73 up- and 18 downregulated) and only 44 in female KO MEFs (27 up- and 17 downregulated, Figure 7). A higher number of Atf-4 targets in male MEFs is expected, and the fact that male- and female-upregulated gene targets show only a 27% overlap supports the sex-specific nature of ISR in Sirt-3 KOs.

*Atf-4* can be induced in the case of ER stress, amino acid deprivation, unfolded protein accumulation, mitochondrial dysfunction, hypoxia, oxidative stress, and other stressors [49,50,51]. These responses overlap and include many commonly affected genes, but to a certain extent, they can be distinguished through gene expression patterns. Thus, ER stress is characterized by *Atf-4*-mediated induction of ER-associated chaperones such as *Hspa-5* [52]. Disturbed metabolism and availability of amino acids induces phosphoserine amino transferase-1 (*Psat-1*) and asparagine synthetase (*Asns*). As a response to hypoxia/oxidative stress, *Atf-4* induces cystathionase (Cth) and heme oxygenase (*Hmox-1,* [53,54]. Additionally, *Atf-4* has been identified as a key regulator of the mitochondrial stress response, along with the canonical UPR^mt^ transcription factor *Atf-5* [44,55,56]. Both the *Atf-4* and *Atf-5* pathways act to rescue mitochondrial homeostasis and the capacity for proper protein folding through the induction of chaperones (*Hspe1, Hspd1* and *Hspa9*) and proteases (*ClpP* and *LonP1*). Table 4 summarizes the differences in the expression of key ISR effectors between male and female KO MEFs.

### 2.5. Sirt-3 KO Induces UPR^ER^ Independent of Sex

Both male and female KO MEFs mounted an endoplasmic reticulum (ER) unfolded protein stress response (UPR^ER^), as shown by the induction of the ER-specific chaperone BiP, protein disulfide isomerases *Pdia-4* and *Pdia-5*, ER-associated protein degradation (ERAD)-involved *Herpud-1* and i. Sirt-3 has been previously implicated in the UPR^ER^ [58]. UPR^ER^ can be activated through three transmembrane misfolded/unfolded protein sensors: inositol requiring kinase 1 (*Ire-1*, mouse *Ern-1*), pancreatic ER eIF2a kinase (*Perk*), and activating transcription factor 6 (*Atf-6*). All of them are kept in an inactive state by bound Hspa-5 (BiP). Misfolded proteins in the ER compete for BiP binding. BiP dissociation activates kinases through dimerization of Ire1, oligomerization of Perk or Atf-6 transport to the Golgi, where the transcriptionally active form is formed by proteolytic cleavage [59]. In KO MEF, the elevation of BiP indicates general UPR^ER^ activation. The activated Ire1 pathway is mediated by Xbp-1, while Perk induces Atf-4 through eIF-2α phosphorylation. *Atf-6* mRNA levels were not changed in KO MEFs, but this does not necessarily reflect the activity of a corresponding pathway. As we detected increased expression of *BiP, Xbp-1, Atf-4*, and a number of their target genes (Table 4), we can conclude that loss of Sirt-3 induces UPR^ER^ through at least two sensor kinases, Perk and Ire1, and independently of sex.

### 2.6. Loss of Sirt-3 Activates the UPR^mt^ Stress Response in Male MEF

To maintain appropriate levels of mitochondrial function, cells can use several stress-response pathways to respond to various aspects of mitochondrial dysfunction. Thus, perturbations in mitochondrial proteostasis activate UPR^mt^ fusion, and fission can be utilized to control or restore the proper abundance, stability or distribution of mitochondria [60,61]. Moreover, irreversibly damaged mitochondria can be removed by selective autophagy (mitophagy). *Atf-4, Atf-5* and *Ddit-3* are major regulators of the response to the mitochondrial unfolded protein stress response [56,62]. While the Atf-4/Chop branch of the UPR^mt^ can be activated in various ways as part of the ISR, Atf-5 localizes to mitochondria, is induced by mitochondrial stress and is translocated to the nucleus. Once imported, it activates the expression of genes involved in mitochondrial quality control, such as chaperones, proteases and antioxidant enzymes, to restore mitochondrial function. Our expression data (Figure 6, Table 4) shows a large increase in *Atf-4, Chop/Ddit-3* and *Atf-5* gene expression exclusively in male KO MEFs. Interestingly, while the mitochondrial chaperone *Hspa9* and *LonP* protease were upregulated, we detected a decrease in *Hspe-1* and *Hspd-1* in male KO MEFs. Female KO MEFs did not show any changes in chaperone/protease expression. Downregulation of *Hspe-1* and *Hspd-1* could indicate a decreased need for chaperones due to low mitochondrial translation. In the case of mitochondrial stress, cells can decrease mitochondrial protein translation until stress resolves. Furthermore, we noticed the reduced expression of mitochondrial ribosomal proteins (Mrps, Table 5). Although Mrps are encoded in the nucleus, translated in the cytoplasm and then imported to mitochondria, their downregulation is related to various diseases, cell cycle progression, metabolic adaptations to stress and apoptosis [63,64]. This finding points to a male-specific Sirt-3-dependent increase in misfolded protein content (LonP increase) followed by suppression of mitochondrial translation, as shown by a decrease in mitochondrial chaperones and ribosomal proteins. Sirt-3 is known to mediate the cellular response to UPR^mt^ induced by overexpression of mutant endonuclease G [65]. Here, we show that, in male KO MEFs, the sole loss of Sirt-3 is sufficient to mount this response. On the other hand, regarding mitochondrial proteostasis, female MEFs either tolerate loss of Sirt-3 or are unable to react properly. Considering the global expression patterns described thus far, the first option is more probable. Finally, we investigated the levels of genes involved in mitophagy and found differences in Bcl-2 interacting protein 3 (*Bnip-3*), Unc-51-like kinase 1/2 (*Ulk1/2*), and neighbor of BRCA1 gene 1 (*Nbr-1*)**,** but the results are not very conclusive; therefore, we did not engage in further analyses.

### 2.7. Male-Restricted ISR Induction as a Consequence of Oxidative Stress

**Figure 8:**
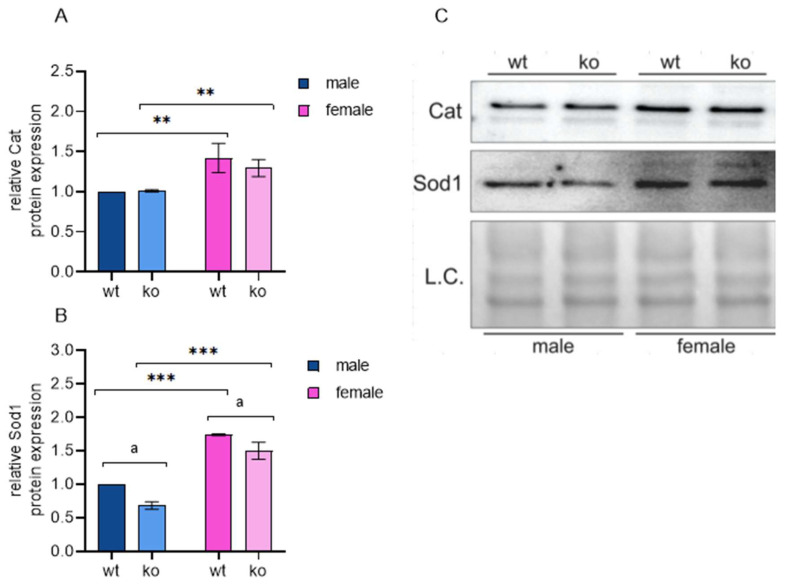
Female MEFs show a genotype-independent increase in superoxide dismutase 1 (Sod-1) and catalase (Cat). (**A**) Graphical display of the averaged densitometry values for Cat protein expression. The main effect of sex: F(1, 4) = 21.968, *p* < 0.01, partial η^2^ = 0.846. ** *p* < 0.01, female WT vs. male WT, female KO vs. male KO. (**B**) Graphical display of the averaged densitometry values for Sod-1 protein expression. The main effect of Sirt-3: F(1, 4) = 27.813, partial η^2^ = 0.874; ^a^ *p* < 0.01, male WT vs. male KO, female WT vs. female KO; the main effect of sex: F(1, 4) = 221.149, partial η^2^ = 0.982. *** *p* < 0.001, female WT vs. male WT, female KO vs. male KO. (**C**) Representative immunoblot of Cat and Sod-1 protein expression. Two-way ANOVA with post hoc Bonferroni correction. L.C., loading control. The results are presented as the mean ± SD.

Regarding oxidative stress, two lines of evidence point to different responses to the loss of Sirt-3 in male and female MEFs. First, as described before, male KO MEFs show a significant Hif-1α response. As the MEFs we used are stable cell lines, male KO MEFs established a steady state stress response due to chronic pseudohypoxia caused by Sirt-3 loss. On the other hand, both WT and KO female MEFs obviously sustain a level of oxidative stress defense sufficient to avoid Hif-1α stabilization. Second, we observed that only male KO MEFs mounted a strong ISR-mediated antioxidant response: in addition to the major ISR inducer *Atf-4* and key redox regulator *Nrf-2*, male KO increased the expression of *Hmox1* (heme oxygenase 1, an antioxidant enzyme and known Nrf-2 target), *Gclc* (glutamate-cysteine ligase; major regulator in glutathione synthesis) and the ER-associated glutathione peroxidase *Gpx-7*. To confirm the differential oxidative state in male and female KO MEFs, we analyzed the protein expression of two main antioxidant enzymes, Sod-1 and Cat. In agreement with several previous studies that showed higher Sod levels in females [66,67,68], we detected increased Sod-1 and Cat protein expression in female cells compared to male cells, independent of Sirt-3 (Figure 8, Supplementary Figures S5 and S6). Other antioxidant enzymes did not show significant sex-specific differences at the mRNA level. These results suggest that female MEFs benefit from their inherently higher Sod-1/Cat levels to compensate for the loss of Sirt-3. On the other hand, male KO MEFs are forced to induce a complex antioxidant response to maintain satisfactory cellular homeostasis levels.

## 3. Discussion

Sirt-3 is most often described as a mitochondrial deacetylase that plays a pivotal role in regulating mitochondrial function and maintaining cellular energy balance. Proteomic analyses have identified hundreds of (de)acetylation sites regulated by Sirt-3 [69], and focused studies are still revealing new Sirt-3 targets. Given the large number of cellular processes in which Sirt-3 is involved, it is likely that it serves as a mediator in fine tuning the cell’s metabolic status and not as an on/off switch. Therefore, Sirt-3 KO mice and cell lines are viable and do not show major anatomical or pathological aberrations. However, at the level of an organism, even slight but persistent metabolic alterations can have long-term effects on the lifespan, healthspan, aging, and development of age-related diseases. Indeed, Sirt-3 has been proposed to increase lifespan in yeast [70] and humans [71]. Decreased Sirt3 levels have been implicated in the development of neurodegenerative disorders such as amyotrophic lateral sclerosis, Parkinson’s disease (PD), Alzheimer’s disease (AD), and probably Huntington’s disease [72,73,74], while its role in cancer is not clear yet [75,76,77]. All these pathologies require time to develop, and the cells experience suboptimal conditions for long periods. Experimentally, chronic Sirt-3 deficiency in our stable Sirt-3 KO cellular model should more closely resemble the effects of inherited or age-related Sirt-3 decrease, as opposed to commonly used conditional or transient KOs. The latter is probably of less biological relevance. Many Sirt-3-related pathologies show different incidences, onsets and/or progression between sexes, such as PD, AD, multiple sclerosis, cardiovascular diseases, and many cancer types. However, most studies ignore sex-specific differences. As a reflection of natural differences in metabolism, response to environmental stimuli and consequential responses at the molecular level, sex should be considered an important parameter in molecular medicine research. Therefore, we aimed to identify male- and female-specific events in response to a common molecular event, i.e., the loss of Sirt-3.

Here, we show that many consequences of Sirt-3 loss are sex-specific. Some of the affected pathways were described before but were not discussed in the context of sex, mostly because of the preferential use of male animals and cell lines or averaging male and female datasets, which is often the case.

Global gene expression difference between Sirt-3 WT and KO MEFs identified several pathways affected differently between the genotypes, but apart from oxidative stress response, the data were not very informative. This is expected due to the inherent diversity of Sirt-3 targets and basal sex-related differences in gene expression, which affect the enrichment results. However, sex-dependent DEG sets supported by protein expression data revealed a striking difference between male and female Sirt-3 KOs. First, a strong Hif-1α response observed in male KO MEFs implies the impaired oxygen reduction in male KO mitochondria, even though oxygen levels were maintained at normal concentrations. Inefficient electron transfer increases mitochondrial ROS, stabilizing Hif-1α through inhibition of prolyl-hydroxylases, which mark Hif-1α for proteasomal degradation. We did not observe a Hif-1α response in female MEFs, which raises several possible explanations: either female MEFs are able to maintain adequate mitochondrial function, or they are more capable of detoxifying excess ROS. We found an increase in Sod-1 and Cat at both the mRNA and protein levels in female MEFs of both genotypes, meaning that they were not induced by Sirt-3 loss; instead, this is a female-specific trait. We therefore hypothesize that the loss of Sirt-3 affects mitochondria similarly in both sexes; however, females benefit from their inherent increased antioxidant protection and are able to maintain ROS levels low enough to avoid a significant response to hypoxia. This is supported by a similar decrease in C1-driven respiration in both sexes. As Sirt-3 is known to activate Sod-2 and Cat [78,79], its loss is expected to affect cellular oxidative status, and female MEFs can compensate through higher expression of Sod-2/Cat. Additionally, since Sirt-3 expression decreases during aging [80], these findings can contribute to sex-inclusive aging biology research. Metabolic effects downstream of Hif-1α also follow a sex-specific pattern: male Sirt-3 KO MEFs increase glycolytic flux and the pentose phosphate pathway, downregulate the TCA cycle and decrease mitochondrial ATP production, as shown by AMPK phosphorylation. Male KO MEFs compensate for their low energy status through the suppression of fatty acid synthesis and an increase in mitochondrial beta-oxidation. Female KO MEFs show none of the metabolic shifts described, suggesting that their mitochondrial function is preserved in the absence of Sirt-3 and the resulting increase in ROS levels. Earlier studies have shown that females are more efficient in ROS detoxification [66,67,68,81,82]. The inability of male KO to efficiently deal with the high ROS production rate induces a broad Atf-4-mediated ISR. ISR can be triggered by various stressors, and we show that in male KO cells ISR is a result of mitochondrial proteotoxic stress. Mitochondrial proteins are damaged by excess ROS, leading to the accumulation of unfolded proteins in the mitochondrial matrix. This branch of the ISR is known as the UPR^mt^ and is not induced in female KO MEFs. We propose that Sirt-3 loss initially leads to decreased activity of OXPHOS components [83]. Inefficient electron transfer results in an increase in ROS, which are efficiently detoxified in female KO MEFs, avoiding further damage. In contrast, male KOs are unable to neutralize ROS at the proper rate, which induces the Hif-1α response with an associated reduction in OXPHOS and accumulation of misfolded and damaged proteins as a secondary effect. OXPHOS is reduced in male KO MEFs also due to lower levels of mitochondrial ribosomal proteins, consequential to global protein synthesis inhibition in ISR. This further supports female Sirt-3 KO-increased antioxidant capacity compared to male MEFs. ISR has been implicated in the pathogenesis of AD, PD, and amyotrophic lateral sclerosis. Chronic activation of the ISR in neurons can lead to neuronal dysfunction, synaptic impairment, and neuroinflammation, contributing to disease progression. Additionally, ISR-mediated dysregulation of proteostasis and autophagy may play a role in the accumulation of the misfolded proteins and protein aggregates characteristic of these diseases. ISR signaling pathway has been implicated in metabolic disorders such as obesity, type 2 diabetes, and non-alcoholic fatty liver disease. Chronic activation of the ISR in response to nutrient excess or ER stress can lead to insulin resistance, inflammation, and lipotoxicity in tissues such as adipose tissue, liver, and pancreas. Also, dysregulation of the ISR can contribute to cellular senescence and inflammation, which are hallmarks of aging and age-related pathologies (reviewed in [84]). Here, we present firm evidence that sex-specific differences must be considered as an indispensable parameter in future research on all these conditions. On the other hand, Sirt-3 deficiency induces UPR^ER^ independent of sex. In this case, females’ advantage in antioxidative capacity does not rescue them from protein damage and misfolding in this compartment, suggesting separate Sirt-3 roles in ER and mitochondrial homeostasis. As knowledge about the role of Sirt-3 in ER stress is limited [85], further research is needed to resolve the background of its sex-dependent and sex-independent functions.

### Perspectives & Significance

The sex-specific differences described in this study are significant and include some crucial cellular processes. While basal protein expression in female MEFs allows them to tolerate lower respiration and high ROS levels after the loss of Sirt-3, male MEFs adapt in a more drastic way by entering a prolonged state of cellular stress. This allows the cells to avoid apoptosis or necrosis but comes at the price of slower growth and inefficient nutrient/oxygen utilization. This study provides new insights into the sex-specific effects of Sirt-3 deficiency and shows that the consequences of Sirt-3 loss differ markedly in male and female cell models. Future studies should aim to define the elements that enable female cells to sustain normal metabolic and oxidative states. Furthermore, as Sirt-3 is considered a potential therapeutic target, our study should encourage the inclusion of sex as a parameter in both fundamental research and preclinical studies. How these findings will impact the discovery of sex-specific aspects of aging and longevity, the etiology and treatment of diseases such as neurodegenerative and cardiovascular diseases and cancer research could be an exciting venue in the near future.

## 4. Materials and Methods

### 4.1. Cell Lines

Thirteen-day-old embryos of Sirt-3 WT (129S1/SvImJ, Stock No: 002448 Jackson Laboratory, Bar Harbor, ME, USA) and Sirt-3 KO (Stock No: 012755, Jackson Laboratory, Bar Harbor, ME, USA) female mice were used for isolation of male and female mouse embryonic fibroblasts (MEFs) according to [86], with the modification that individual embryos were isolated to enable sex-based differentiation. Cells were maintained in a 37 °C incubator with 5% CO_2_ and immortalized by stable transfection with SV40 T-antigen-containing plasmid. Sex determination of MEFs was performed by real-time PCR analysis utilizing specific primers targeting the sex-determining region Y (Sry) gene on the Y chromosome. For both RNA extraction and Western blots, low-passage cells were used.

### 4.2. RNA Sequencing

MEFs of both genotypes and sexes were seeded into 6-well plates at 4 × 10^5^ cells per well in triplicate. After 48 h, RNA was extracted using a Qiagen RNeasy mini kit. RNA samples were sent to a commercial NGS service provider (Novogene, Cambridge, UK) for sample quality control, library prep, and sequencing at targeted 15 million reads per sample. RNA quality was assessed using an Agilent Bioanalyzer. Following polyA enrichment, RNA was fragmented, and first-strand cDNA was synthesized using random hexamer primers followed by second-strand cDNA synthesis. After end repair, A-tailing, adapter ligation, size selection, amplification, and purification, libraries were normalized and paired-end sequenced on an Illumina NovaSeq 6000 sequencer (Illumina, San Diego, CA, USA).

### 4.3. Western Blot Analysis

The proteins were isolated from MEFs as described previously [87]. Proteins at a concentration of 20 μg/μL were separated by SDS-PAGE and subsequently transferred onto a PVDF membrane (Roche, Basel, Switzerland). After blocking, the membranes were incubated overnight at 4 °C with the following primary antibodies: Hif-1α, Cell Signaling Technology D2U3T #14179; catalase, Abcam ab16731; superoxide-dismutase 1, Abcam ab16831; fatty acid synthase, Santa Cruz sc-48357; trifunctional enzyme subunit beta, mitochondrial, Hadhb, Santa Cruz sc-271496; phospho-AMPK (Thr172), phosphorylated AMP-induced protein kinase, Cell Signaling (40H9). An appropriate horseradish peroxidase (HRP)-conjugated secondary antibody was utilized for chemiluminescence detection. Total protein normalization was achieved using AmidoBlack (Sigma Aldrich, St. Louis, MO, USA). Immunoblots were detected using the Alliance 4.7 Imaging System (UVITEC, Cambridge, UK) and an enhanced chemiluminescence kit (Thermo Fischer Scientific, Waltham, MA, USA). Experiments for each protein of interest were performed at least two times.

### 4.4. Oxygen Consumption

The cells were trypsinized, and then kept on ice unless otherwise specified. Respiration buffer (150 mM KCl, 1 mM EGTA, 20 mM TRIS, 5 mM KH2PO4, pH 7.4) was used with the cells for the determination of oxygen consumption. The cells (5 × 10^6^) resuspended in 50 µL of cell culture media were added into 450 µL respiration buffer in the Clarks type electrode chamber (Oxygraph, Hansatech Instruments Ltd., Pentney, UK). Solely the plasma membrane was permeabilized by adding 0.01% (*w*/*v*) digitonin. For complex 1 assessment, cells were incubated with 2.5 mM glutamate and 1.25 mM malate. Mitochondrial respiration was accelerated by the addition of ADP (500 µM final concentration) for state 3 respiration measurements. Oxygen consumption was calculated in nmol/min/number of cells.

### 4.5. RNA-seq Data Analysis

Adapter sequences and low-quality base trimming were performed in Novogene. The number of reads per gene (reference genome GRCm38) was calculated using Salmon 1.10.2 [88], and a TPM (transcripts per million) matrix was used for differential gene expression analysis. DeSeq2 with the Wald test and parametric fit was used either as an R package or through the web-based tool RaNa-Seq (https://ranaseq.eu/index.php, accessed on 10 January 2024). The adjusted *p* value threshold was set to 0.05. RaNa-Seq also generates sample clustering heatmaps, PCA and functional analysis, such as gene set enrichment analysis (GSEA). For pathway enrichment, we used the open source tool Enrich [57]. Gene sets corresponding to individual pathways were downloaded from Harmonizome 3.0 [89].

### 4.6. Statistical Analysis

For the statistical analysis of Western blot data, SPSS for Windows (v.17.0, IBM, Armonk, NY, USA) was used. A two-way ANOVA was performed to reveal the interaction effect of Sirt-3 and sex. If a significant interaction was observed, a Bonferroni adjustment was made to correct for multiple comparisons within each simple main effect separately. Significance was set at *p* < 0.05.

## 5. Conclusions

1. Male and female MEFs mount significantly different adaptive metabolic responses to loss of Sirt3 function and the resulting mitochondrial dysfunction;
2. Female MEFs compensate for Sirt-3 loss, at least in part, through increased antioxidant enzyme expression; in male cells, Sirt3 knock-out induces pseudohypoxia resulting in chronic oxidative stress, shift to glycolysis and fatty acid oxidation;
3. In male MEFs, loss of Sirt3 increases Hif-1a and induces an ATF4-mediated integrated stress response;
4. In the MEF (hormone-independent) experimental model, female cells exhibit a higher level of protection from oxidative stress induced by impaired mitochondrial function, maintaining the normal metabolic and oxidative states;
5. Through maintenance of mitochondrial function, Sirt3 is involved in aging and neurodegenerative and cardiovascular diseases; significant differences between sexes should be accounted for in future research.

## Figures and Tables

**Table 1:** Top pathways significantly enriched for male and female Sirt-3 KO common DEGs.

Pathway	False Discovery Rate
Focal adhesion	2.22 × 10^−10^
Focal adhesion: PI3K-Akt-mTOR signaling pathway	4.79 × 10^−9^
Mechanisms associated with pluripotency	0.00047
Cholesterol metabolism with Bloch and Kandutsch–Russell pathways	0.00088
Comprehensive IL-17A signaling	0.00088
Adipogenesis genes	0.0029
ESC pluripotency pathways	0.0031
Mapk signaling pathway	0.0037
Integrin-mediated cell adhesion	0.0037
Alpha 6 beta 4 integrin signaling pathway	0.0111
TGF-beta signaling pathway	0.0118
Retinol metabolism	0.0242
Wnt signaling pathway and pluripotency	0.0318
Omega-9 fatty acid synthesis	0.0464
Matrix metalloproteinases	0.0464
Oxidative stress and redox pathway	0.0464

**Table 2:** Selected KEGG 2021, WikiPathway 2021, Reactome 2022 and Panther 2016 pathways enriched in male or female Sirt-3 KO-specific DEGs.

Male MEF	Female MEF
EGF receptor signaling pathway	De novo pyrimidine ribonucleotides biosynthesis
Pentose phosphate pathway	De novo purine biosynthesis
Wnt signaling pathway	Sterol regulatory element-binding proteins (SREBP) signaling
Cadherin signaling pathway	Mitogen activated protein kinase kinase/MAP kinase cascade
FGF signaling pathway	Ras pathway
Glycolysis	PI3 kinase pathway
Hypoxia response via HIF activation	
De novo purine biosynthesis	
PI3 kinase pathway	
JAK/STAT signaling pathway	

**Table 3:** Expression of glycolytic, TCA cycle, pentose-phosphate pathway and fatty acid metabolism-related genes in male and female Sirt-3 KO relative to WT MEFs.

Pathway	Gene	Log_2_ Fold Change—Male KO MEF	Adjusted *p*-Value—Female KO MEF	Log_2_ Fold Change—Female KO MEF	Adjusted *p*-Value—Female KO MEF
	*Prkaa2 (AMPK)*	**0.57**	**1.10 × 10^−4^**	−0.248	0.26
**Glycolysis and TCA cycle**	*Lactate dehydrogenase B (Ldhb)*	**2.09**	**5.60 × 10^−7^**	0.16	0.33
	*Mitochondrial pyruvate carrier 1 (Mpc1)*	**0.37**	**0.02**	0.15	0.82
	*Pyruvate dehydrogenase E1 alpha 1 subunit (Pdha1)*	0.14	4.40 × 10^−4^	0.18	4.40 × 10^−6^
	*Phosphofructokinase-1. Liver-type (Pfkl)*	0.4	1.60 × 10^−7^	0.27	1.20 × 10^−9^
	*Phosphofructokinase 1 muscle-type (Pfkm)*	**0.34**	**3.40 × 10^−4^**	0.07	0.23
	*Phosphofructokinase 2 (Pfkfb2)*	**0.48**	**0.012**	−0.11	0.86 Ima ima
	*Pyruvate kinase (Pkm)*	**−0.11**	**0.016**	0	0.27
	*Aldolase A (Aldoa)*	0.44	0.694	**−0.15**	**3.00 × 10^−4^**
	*Phosphoglycerate kinase (Pgk1)*	0.02	0.624	**0.14**	**5.00 × 10^−6^**
	*Isocitrate dehydrogenase (Idh2)*	0.2	4.60 × 10^−5^	0.11	0.03
	*Pyruvate dehydrogenase kinase isoenzyme 1 (Pdk1)*	1.23	1.00 × 10^−7^	0.63	0.002
	*Succinate dehydrogenase complex subunit C (Sdhc)*	0.03	0.004	0.03	0.038
	*Alpha-ketoglutarate dehydrogenase (Ogdh)*	0.05	0.152	0.05	0.264
	*Succinyl-CoA ligase [ADP-forming] subunit beta (Sucla2)*	0.08	0.382	0.1	0.414
	*Succinate dehydrogenase (Sdha)*	−0.12	0.729	−0.04	0.593
	*Fumarate hydratase (Fh1)*	0.03	0.926	0.12	0.363
	*Malate dehydrogenase 1 (Mdh1)*	−0.02	0.528	0.16	0.191
	*Malate dehydrogenase 2 (Mdh2)*	−0.04	0.870	0.01	0.666
	*Citrate synthase (Cs)*	−0.07	0.530	−0.04	0.947
	*Pyruvate carboxylase (Pcx)*	−0.04	0.998	−0.16	0.790
	*Aconitase (Aco1)*	0.05	0.465	0.02	0.940
**Pentose-phosphate pathway**	*Glucose-6-Phosphate Dehydrogenase (G6pdx)*	**0.22**	**4.10 × 10^−5^**	0.21	0.124 Ima ima
	*6-Phosphogluconolactonase (Pgls)*	0.28	2.10 × 10^−4^	0.18	0.01
	*6-Phosphogluconate Dehydrogenase (Pgd)*	**0.17**	**4.90 × 10^−4^**	−0.02	0.947
	*Ribulose-5-Phosphate Isomerase (Rpia)*	−0.1	0.922	−0.08	0.947
	*Transketolase (Tkt)*	**0.14**	**7.80 × 10^−6^**	0.03	0.380
	*Transaldolase (Taldo1)*	−0.07	0.718	−0.08	0.79
**Fatty acid metabolism**	*Acetyl-coA carboxylase (Acaca)*	−0.263	0.093	0.056	0.008
	*Slc27a4*	**0.227**	**4.4 × 10^−4^**	0.007	0.947
	*Fasn (Fatty acid synthase)*	**−0.134**	**0.011**	0.039	0.940

**Table 4:** Expression of selected ISR effectors in male and female Sirt-3 KO MEFs relative to Sirt-3 WT MEF. Log_2_ fold and *p*_adj_ values for genes showing significant expression changes are in bold (*p*_adj_ < 0.05).

Gene	Log_2_ Fold Change—Male KO MEF	Adjusted *p*-Value—Female KO MEF	Log_2_ Fold Change—Female KO MEF	Adjusted *p*-Value—Female KO MEF	Function
*Hspd1*	**−0.16**	**3.30 × 10^−5^**	−0.02	0.72	Mitochondrial chaperones
*Hspe1*	**−0.267**	**2.30 × 10^−6^**	0.02	0.75	
*Hspa9*	**0.071**	**0.021**	**−0.121**	**5.5 × 10^−4^**	
*Hspa-5 (BiP)*	**0.1**	**9.80 × 10^−14^**	**0.2**	**9.00 × 10^−27^**	ER chaperones
*Calr*	**0.16**	**1.30 × 10^−8^**	**0.31**	**4.50 × 10^−61^**	
*Pdia4*	**0.3**	**4.20 × 10^−15^**	**0.26**	**2.10 × 10^−15^**	
*Pdia5*	**0.37**	**1.70 × 10^−10^**	**0.32**	**4.40 × 10^−9^**	
*Herpud1*	**0.87**	**1.10 × 10^−26^**	**0.4**	**0.002**	
*Derl1*	**0.364**	**3.1 × 10^−11^**	0.111	0.053	
*ClpP*	0.028	0.922	−0.06	0.947	Proteases
*LonP*	**0.295**	**1.8 × 10^−13^**	**−0.19**	**2.0 × 10^−4^**	
*Cth*	**0.974**	**1.4 × 10^−12^**	**−0.711**	**3.1 × 10^−8^**	Atf-4 mediated response to hypoxia/oxidative stress
*Hmox1*	**0.494**	**6.5 × 10^−11^**	−0.202	0.082	Heme Oxygenase 1, antioxidant enzyme and a known Nrf-2 (Nfe2l2) target
*Gclc*	**0.168**	**0.01**	−0.144	0.346	Glutamate-cysteine ligase; glutathione synthesis
*Asns*	**0.836**	**1.2 × 10^−55^**	0.023	0.728	Atf-4 mediated amino acid metabolism and transport
*Psat1*	**0.279**	**1.2 × 10^−19^**	−0.052	0.941	Phosphoserine aminotransferase 1, involved in serine biosynthesis
*Slc3a2*	**0.304**	**2.0 × 10^−10^**	−0.084	0.071	Amino acid transporter; ER stress regulator [57]

**Table 5:** Expression of mitochondrial ribosomal proteins in male and female Sirt-3 KO MEFs relative to WT MEFs. Log_2_ fold and *p*_adj_ values of significantly downregulated genes are in bold (*p*_adj_ < 0.05).

Gene	Log_2_ Fold Change—Male KO MEF	Adjusted *p*-Value—Female KO MEF	Log_2_ Fold Change—Female KO MEF	Adjusted *p*-Value—Female KO MEF	Function
*Mrpl1*	**−0.269**	**0.045**	**−0.225**	**0.049**	Mitochondrial large unit ribosomal protein
*Mrpl9*	**−0.275**	**0.037**	−6.2 × 10^−5^	0.947	Mitochondrial large unit ribosomal protein
*Mrpl19*	**−0.068**	**0.003**	0.235	0.993	Mitochondrial large unit ribosomal protein
*Mrpl23*	**−0.941**	**0.008**	0.029	1	Mitochondrial large unit ribosomal protein
*Mrpl27*	**−0.334**	**0.037**	−0.075	0.917	Mitochondrial large unit ribosomal protein
*Mrpl38*	**−0.371**	**0.001**	−0.105	0.66	Mitochondrial large unit ribosomal protein
*Mrpl45*	**−0.271**	**0.009**	**−0.240**	**0.036**	Mitochondrial large unit ribosomal protein

## Data Availability

Raw data in fastq.gz format and the TPM table for all samples are deposited and available at https://www.ncbi.nlm.nih.gov/geo/query/acc.cgi?acc=GSE246699, accessed on 31 October 2023. Reviewer secure token: czeveesibtiftgd.

## Supplementary Materials

The following supporting information can be downloaded at: https://www.mdpi.com/article/10.3390/ijms25073868/s1. Reference [90] is cited in the supplementary file.
