# Supplementary material for: Gene Expression Profiling Reveals Fundamental Sex-Specific Differences in SIRT3-Mediated Redox and Metabolic Signaling in Mouse Embryonic Fibroblasts
Source: Int J Mol Sci. 2024 Mar 30;25(7):3868. doi: 10.3390/ijms25073868 (PMC11012119; doi:10.3390/ijms25073868)
Supplement: Supplementary file 1 [file ijms-25-03868-s001.zip › ijms-2914705-supplementary.pdf]

SUPPLEMENTARY FILE

Gene expression profiling reveals fundamental sex-specific differences in SIRT3-mediated redox and metabolic signaling in mouse embryonic fibroblasts

Robert Belužić[1*#], Ena Šimunić[1#], Iva I. Podgorski[1], Marija Pinterić[1], Marijana Popović Hadžija[1], Tihomir Balog[1], Sandra Sobočanec[1]

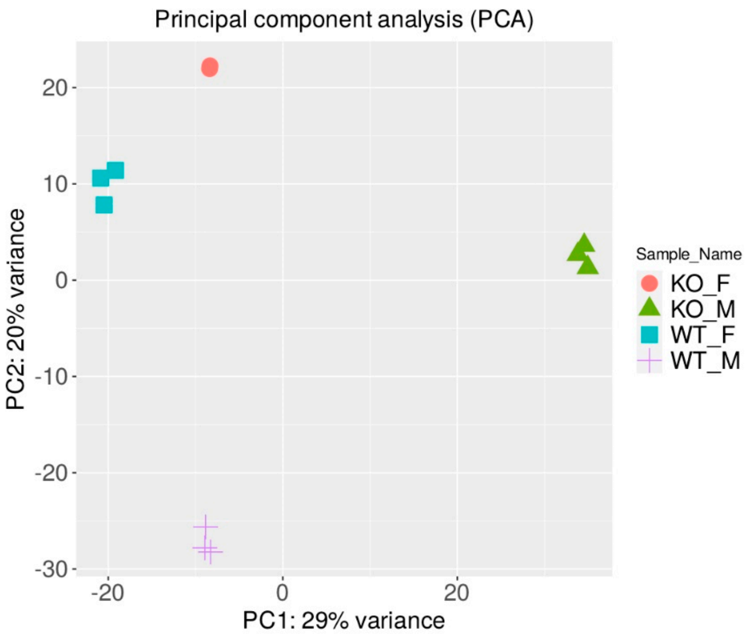

Figure S1. Principal component analysis was performed using IDEP (1) with TPM matrix as input. KO - knockout; WT – wild type; M - male; F-female.

Table S1. TPM values of main ISR regulators and effectors.

	TPM (WT_M)	TPM (KO_M)	P_{adj}_males	TPM (WT_F)	TPM (KO_F)	P_{adj}_females
Hif1a	57.67	83.72	[a]6.80E-27	57.58	60.47	0.244
Atf4	210.72	372.88	[a]4.90E-95	284.00	260.00	[c]0.041
Atf5	208.14	323.73	[a]8.20E-41	206.00	200.00	0.874
Gadd34	38.67	37.98	0.984	42.72	32.51	[a]6.10E-4
Nfe2l1	114.15	134.15	[a]3.20E-11	125.00	132.00	1
Nfe2l2	48.86	55.28	[c]0.01	44.20	45.36	0.896
Xbp1	65.86	90.53	[a]1.40E-13	59.30	84.20	[a]3.80E-14
Chop/Ddit 3	81.31	102.72	[b]0.001	82.09	74.59	0.791
Ddit4	40.07	62.39	[a]2.50E-14	44.70	56.20	[a]1.60E-4

P_{adj} – the marks [a, b, c] are shown in Fig. 5 and 6

<u>Original unedited Western blot images (Figure 2S, 3S, 4S, 5S, 6S)</u>

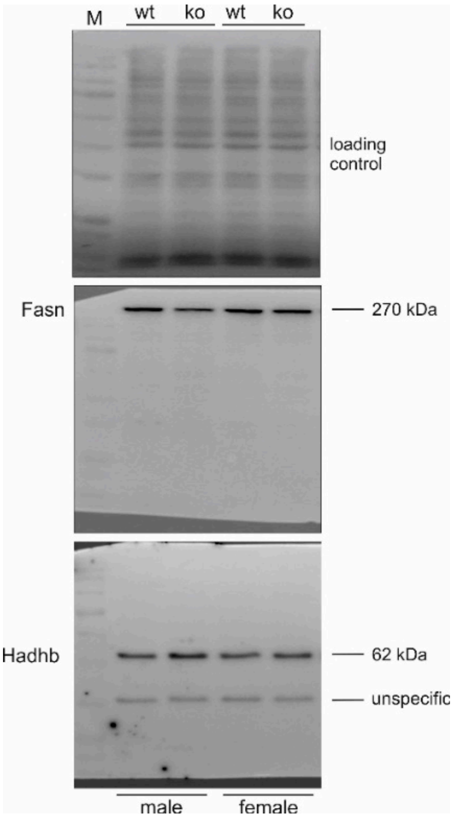

Figure S2. Western blot of Fasn and Hadhb in Sirt3 WT and Sirt3 KO MEF of both sexes. Amidoblack was used as a loading control; 20 micrograms of proteins per lane.

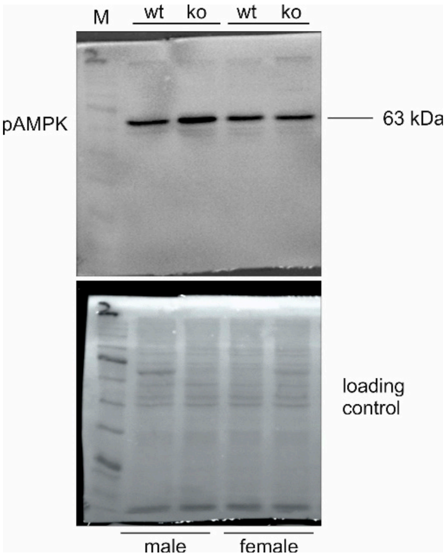

Figure S3. Western blot of pAMPK in Sirt3 WT and Sirt3 KO MEF of both sexes. Amidoblack was used as a loading control; 20 micrograms of proteins per lane.

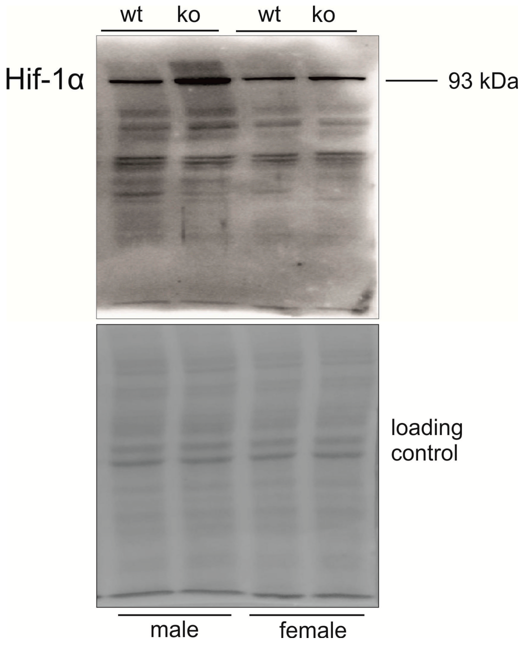

Figure S4. Western blot of Hif-1α in Sirt3 WT and Sirt3 KO MEF of both sexes. Amidoblack was used as a loading control; 20 micrograms of proteins per lane.

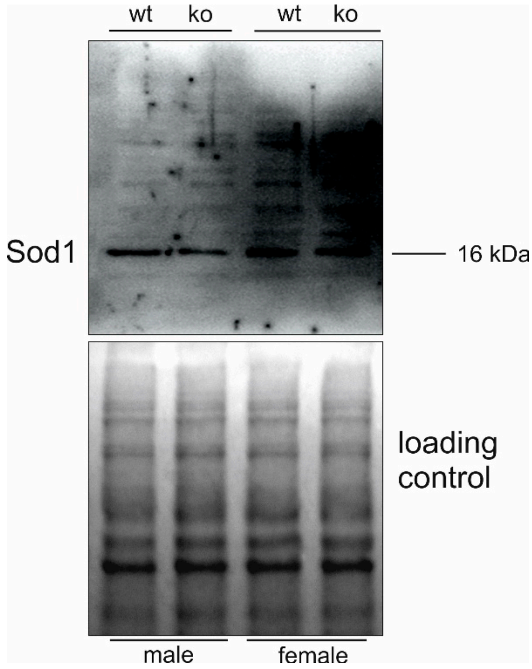

Figure S5. Western blot of Sod1 in Sirt3 WT and Sirt3 KO MEF of both sexes. Amidoblack was used as a loading control; 20 micrograms of proteins per lane.

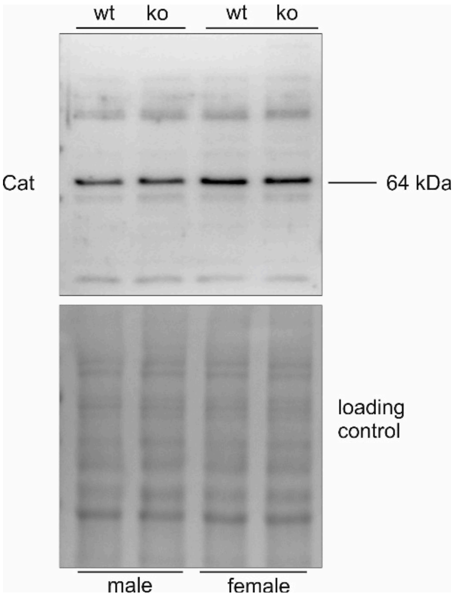

Figure S6. Western blot of Cat in Sirt3 WT and Sirt3 KO MEF of both sexes. Amidoblack was used as a loading control; 20 micrograms of proteins per lane.

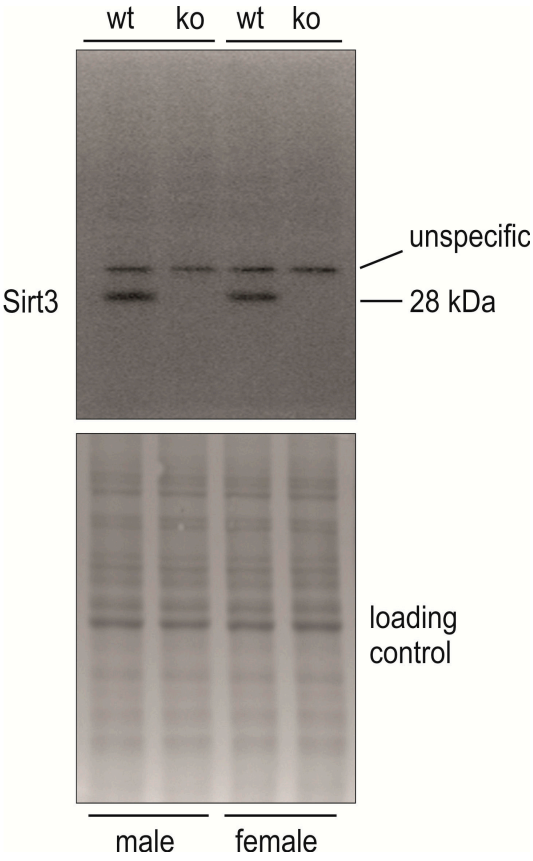

Figure S7. Western blot of Sirt in Sirt3 WT and Sirt3 KO MEF of both sexes. Amidoblack was used as a loading control; 20 micrograms of proteins per lane.

References:

1. Ge S.X.; Son, E.W.; Yao, R. iDEP: An integrated web application for differential expression and pathway analysis of RNA-Seq data. *BMC Bioinform.* **2018**, *19*, 534.
